# Self-cleanable, waterproof, transparent, and flexible Ag networks covered by hydrophobic polytetrafluoroethylene for multi-functional flexible thin film heaters

**DOI:** 10.1038/s41598-019-53243-w

**Published:** 2019-11-13

**Authors:** Ji-Eun Lee, Han-Ki Kim

**Affiliations:** School of Advanced Materials Science and Engineering, Sungkyunkwan University, 2066, Seobu-ro, Jangan-gu, Suwon-si, Gyeonggi-do 16419 Republic of Korea

**Keywords:** Engineering, Materials science

## Abstract

We demonstrate a self-cleanable, waterproof, highly transparent, and flexible Ag network covered by a very thin transparent polytetrafluoroethylene (PTFE) layer using typical magnetron sputtering for multi-functional flexible thin film heaters used in smart windows. By passivation of the self-assembled Ag network with very thin PTFE films, we fabricated a multi-functional Ag network suitable for flexible thin film heaters. At a PTFE thickness of 10 nm, the Ag network passivated by hydrophobic PTFE layer showed a low sheet resistance of 11.64 Ohm/square, high optical transmittance of 80.20% at a wavelength of 550 nm, and high contact angle of 102.42°. In addition, sputtering of the PTFE layer on the Ag network improved the mechanical flexibility and reliability of the Ag network electrode. The flexible and transparent thin film heater (TTFH) with Ag network electrode covered by PTFE layer showed a saturation temperature of 120 °C at low voltage of 4.5 V and power of 2.45 W, as well as a hydrophobic surface suited for self-cleaning smart windows. These multi-functional performances of TTFH indicate that the Ag network/PTFE film-based flexible TTFH could be used as self-cleanable, waterproof TTFHs for curved smart windows in smart buildings and automobiles.

## Introduction

For the better life in building interiors and automobiles, the development of multi-functional smart windows with transparent displays, transparent sensors and electronics, semi-transparent photovoltaics, electrochromic devices, heat generators, and heat-shielding films is necessary to control indoor conditions to afford more comfortable conditions^1–7^. In particular, eco-friend and energy efficient smart windows combined with the Internet of Things to communicate with human indoors are required for next-generation smart buildings and automobiles. Because the portion of windows in recent buildings and automobiles has rapidly increased, the development of multi-functional smart windows critically influences the environment of building and automobile interiors^8,9^ .Among several functions of the smart windows, the transparent thin film heater (TTFH) to remove frost and ice on the surface of window is very important^10,11^. In particular, the TTFHs require hydrophobic surface for the self-cleaning of the smart window, and good flexibility for the curved surface of windows. Therefore, the development of multi-functional films that can be used as heat generators in smart windows is imperative. Because the heat generation in TTFH mainly occurs in transparent conducting electrodes (TCEs), the design of high quality TCE materials could affect the performance of TTFHs^12,13^. Although most TTFHs have been fabricated on Sn-doped In_2_O_3_ (ITO) and F-doped SnO_2_ (FTO) films, they have the critical problems of poor flexibility and hydrophilic surface^14–19^. In our previous work, we report on the feasibility of random mesh-like Ag network as a promising candidate for typical ITO and FTO electrodes for flexible organic solar cells and flexible thin films heaters^20,21^. In addition, as alternatives to oxide-based TCEs, several promising materials have been suggested as transparent electrodes for the TTFHs. Gupta *et al*. reviewed the advance of TCE materials, such as metal oxide film, carbon nanostructure, metal nanowire, metal mesh, and hybrid electrodes for visibly transparent heaters^22^. Yoon *et al*. reported the potential of single-wall carbon nanotube as a flexible TCE for TTFHs, and demonstrated their rapid thermal response and stable reversible heating performance^23^. In addition, Kang *et al*. demonstrated high-performance graphene-based TTFHs with low operation voltage^24^. The Ag nanowire, Ag metal mesh, and Ag network films prepared by a solution-based coating process were employed as TCE for TTFHs^17,18,25,26^. Recently, our group also reported the high performance of TTFHs fabricated on Ag nanowire, Ag metal network, CuO_x_-Cu-CuO_x_ grid, W-doped In_2_O_3_, ITO-Ag-ITO, ZnSnO-AgPdCu-ZnSnO, and MoO_x_-Ag-MoO_x_ electrodes^12,13,27–31^. Although several TCE materials (carbon-based electrodes, metal-based electrodes, and oxide-based electrodes, and hybrid materials) including bare Ag network have been applied in TTFHs, detailed investigation of the self-assembled Ag network passivated by a polytetrafluoroethylene (PTFE) layer for heat generation is still lacking^28,30,32^.

Herein, we report on the characteristics of the self-assembled Ag network covered by a sputtered PTFE layer to apply to flexible heat-generating films. By sputtering of the PTFE passivation layer, we can fabricate hydrophobic and flexible TTFHs for self-cleanable and curved smart windows. The electrical, optical, morphological, and mechanical properties of the Ag network/PTFE films were investigated in detail. Furthermore, we demonstrated the feasibility of Ag network/PTFE films for hydrophobic TTFHs for multi-functional smart windows used in smart buildings and automobiles.

## Results

**Figure 1:**
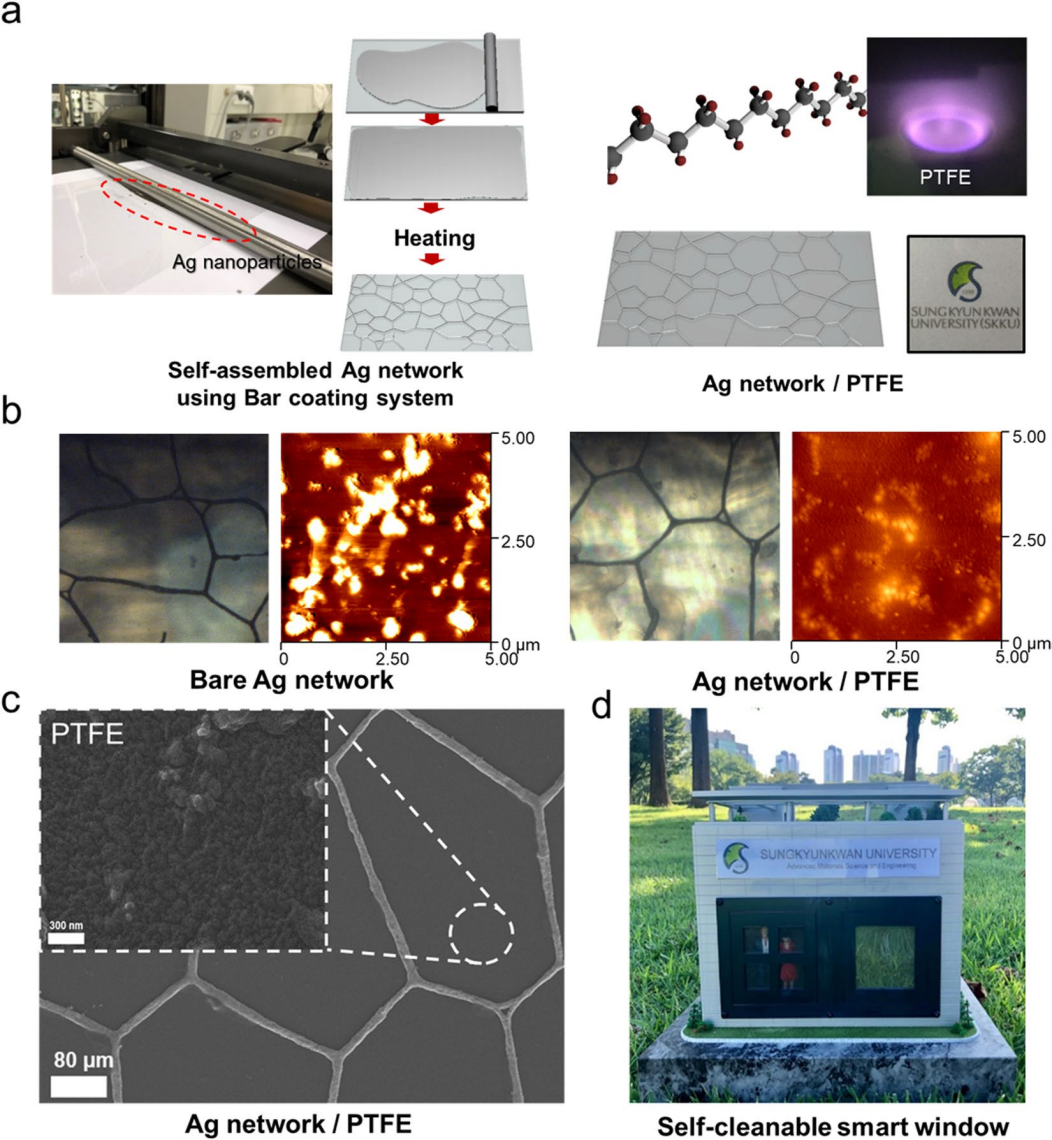
(**a**) Schematics of the fabrication process of the self-assembled Ag network using a bar coating system. The right side shows the structure of the PTFE and RF-sputtered PTFE layer onto Ag network. (**b**) Surface optical microscope and AFM images of bare Ag network and Ag network/PTFE films. (**c**) Surface FESEM image of an intaglio type Ag network passivated by sputtered PTFE film. The enlarged image was obtained from the open space region of the Ag network after sputtering of the PTFE layer. (**d**) Multi-functional Ag network/PTFE films attached on smart window for building model.

Figure 1a shows the fabrication process of the self-assembled Ag network-based multi-functional films on PET substrate for smart windows. First, the self-assembled Ag network was fabricated by a bar-coating process, by using special Ag nanoparticles ink on pre-treated PET substrate mounted on the heating stage of a bar-coater^20^. Before the bar-coating of Ag nanoparticle ink, the surface of the PET substrate was pre-treated by 3-aminopropyltriethoxysilane (C_9_H_23_NO_3_Si) with 1% acetone solution. The pre-treated PET substrate was placed on a heating plate, and a linear bar was subsequently pulled over the Ag nanoparticle ink and PET substrate, as shown in Fig. 1a. The bar-coated sample was annealed at a temperature of 150 °C to form a self-assembled Ag network. After fabrication of the self-assembled Ag network, a hydrophobic PTFE cover layer was sputtered on the Ag network by using MF magnetron sputtering. The sputtered PTFE films uniformly passivated the Ag network, as illustrated on the right side of Fig. 1a. Figure 1b show the opical microscope (OM) and surface AFM images of bare Ag network films and Ag network/ PTFE films. The Ag network/PTFE films showed more smooth surface with an rms value of 4.66 nm than bare Ag network films with rms value of 23.01 nm. Moreover, enlarged FESEM image shown in Fig. 1c from the open space region in the Ag network shows the typical morphology of the PTFE layer, uniformly covering the open space of the Ag network. Using this PTFE passivation layer covered Ag network electrode, we can fabricate the self-cleanable, transparent, flexible, and high-performance thin film heater for use in smart windows, as in Fig. 1d.

**Figure 2:**
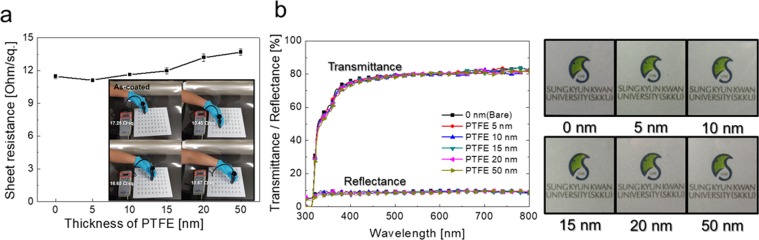
(**a**) Sheet resistance of the Ag network/PTFE films measured *in situ* by four-point probe as a function of the PTFE thickness from (0 to 50) nm. (**b**) Optical transmittance and reflectance of the Ag network/PTFE films at a visible wavelength region with increasing sputtered PTFE thickness. The panels at right show the color and transparency of the bare Ag network and Ag network/PTFE films with different PTFE thickness.

Figure 2a shows the sheet resistance of the Ag network/PTEF films measured *in situ* by four-point probe. Due to the well-connected Ag network as expected in Fig. 1b, the Ag network/PTFE film showed a low sheet resistance with small standard error range. With increasing thickness of PTFE passivation layer, the Ag network/PTFE film exhibited slightly increased sheet resistance from (11.12 to 13.73) Ohm/square ((5 to 50) nm)). Due to the micro-scale height of the Ag network, the Ag network/PTFE films were not significantly affected by the thickness of the PTFE passivation layer. Figure 2b showed an optical transmittance and a reflectance oft he bare Ag network and Ag network/PTFE samples. All the Ag network/PTFE films showed similar transmittance of (79.37–80.17) % and reflectance of (8.42–9.36) % to the bare Ag network, due to the high transparency of the sputtered PTFE layer. The color and transparency of the Ag network/PTFE film are also similar those of the bare Ag network film. Therefore, the existence of the sputtered PTFE layer did not affect the electrical and optical properties of the Ag network electrodes. Although sputtering of the PTFE passivation layer didn’t influence the electrical and optical properties of the Ag network films, the surface energy of the Ag network could be dramatically changed by the covering PTFE layer, consisting of strong chemical C–F bonding^33,34^. The low surface energy of the sputtered PTFE layer could be used as a self-cleanable hydrophobic surface of the Ag network/PTFE films-based thin film heater. To confirm the hydrophobic properties of the Ag network/PTFE films for use as a self-cleaning heat generator, we measured the water contact angle (θ) and calculated the surface energy using a contact angle measurement. Generally, the contact angle on a specific surface could be expressed by Young’s equation, as below:

1 $$\cos \,\theta =({\gamma }_{F}\,-\,{\gamma }_{FL})/{\gamma }_{L}$$

The equation components presented the interfacial free energies of the film-vapor (*γ*_*F*_), film-liquid (*γ*_*FL*_) and liquid-vapor (*γ*_*L*_) interfaces.

**Figure 3:**
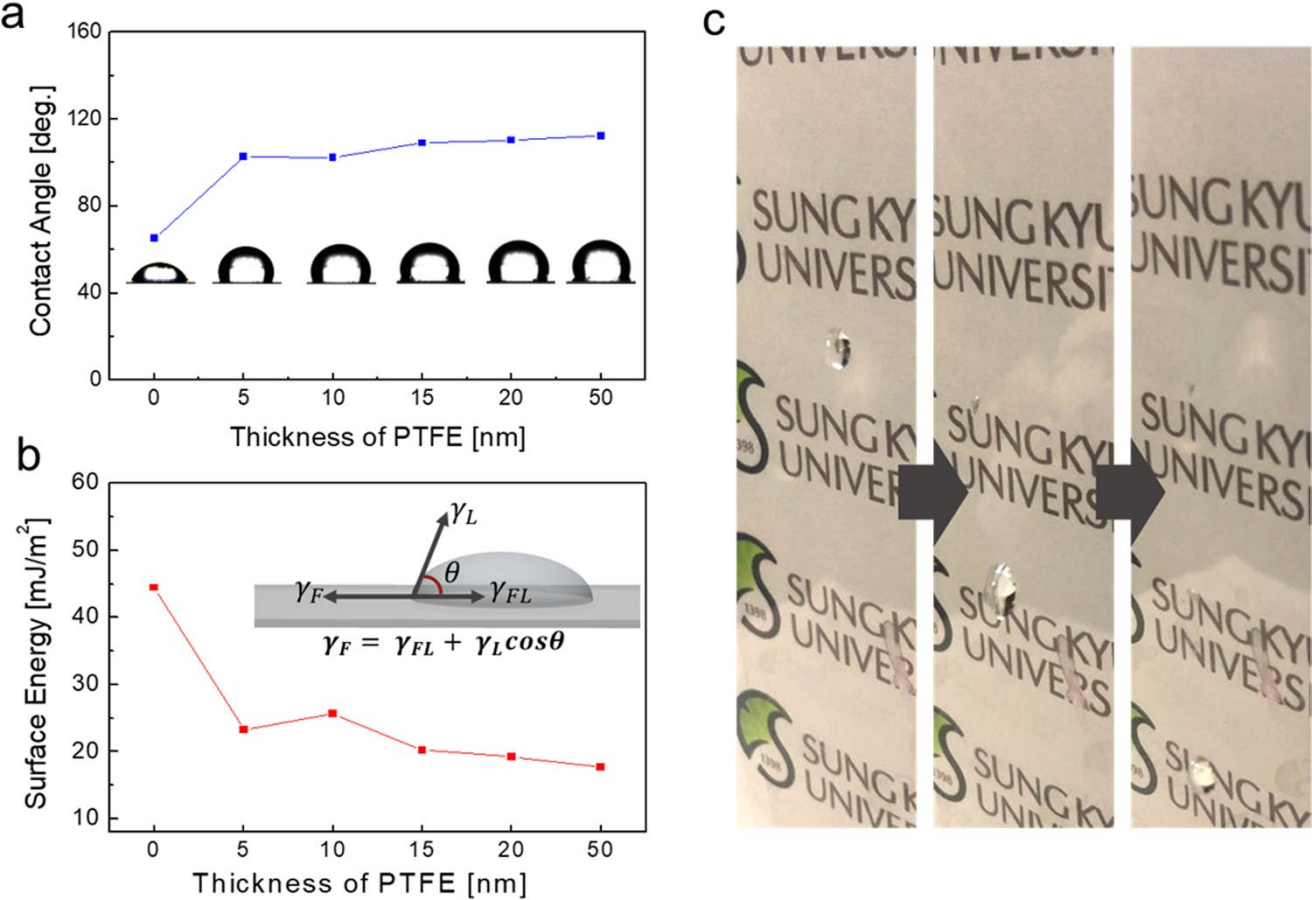
(**a**) Contact angle of the Ag network/PTFE films with increasing sputtered PTFE thickness with inset of water droplet on the surface of Ag network/PTFE films. (**b**) Surface energy of Ag network/PTFE films as a function of PTFE thickness. Inset shows the contact angle of water droplet on the Ag network/PTFE surface. (**c**) Picture of the hydrophobic surface of the Ag network/PTFE films for thin film heaters.

Figure 3a Contact angle exhibited on the pure Ag network and Ag network/PTFE films with increasing PTFE thickness from (5 to 50) nm. Compared to the contact angle of 65.05° of the bare Ag network, the Ag network/PTFE films showed significantly increased contact angle greater than 100°, regardless of the PTFE thickness. The water droplet shape on the surface of the Ag network/PTFE films indicates the formation of hydrophobic surface caused by the sputtered PTFE layer. Furthermore, we calculated the surface energy using the Owens, Wendt, Rabel, and Kaelble (OWRK) method^35^. To calculate the surface energy, two types of solution that had polar (deionized water) and dispersion (diiodomethane) properties were employed. Each component of the surface energy was calculated according to the Fowkes approximation:^36^

2 $${\gamma }_{L}={{\gamma }_{L}}^{d}+{{\gamma }_{L}}^{p}$$

where, the p and d refer to the hydrogen bonding and dispersion force components, respectively:

3 $${\gamma }_{FL}={\gamma }_{F}+{\gamma }_{L}-2\sqrt{{{\gamma }_{F}}^{d}{{\gamma }_{L}}^{d}}$$

4 $$1+\,\cos \,\theta =2\sqrt{{{\gamma }_{F}}^{d}}(\frac{\sqrt{{{\gamma }_{L}}^{d}}}{{\gamma }_{L}})$$

The values of ${{\gamma }_{L}}^{d}$ used are from a table that has already been published for various liquids, and the approximate ${{\gamma }_{F}}^{d}$ value can be known from the contact angle θ where only dispersion forces operate, by using Eq. (4)^37^. In the case of both forces operating (polar and dispersion forces), Eq. (4) can express the general form shown as Eqs. (5) and (6):

5 $${\gamma }_{FL}={\gamma }_{F}+{\gamma }_{L}-2\sqrt{{{\gamma }_{F}}^{d}{{\gamma }_{L}}^{d}}-2\sqrt{{{\gamma }_{F}}^{p}{{\gamma }_{L}}^{p}}$$

6 $$1+\,\cos \,{\rm{\theta }}=2\sqrt{{{{\rm{\gamma }}}_{{\rm{F}}}}^{{\rm{d}}}}(\frac{\sqrt{{{{\rm{\gamma }}}_{{\rm{L}}}}^{{\rm{d}}}}}{{{\rm{\gamma }}}_{{\rm{L}}}})+2\sqrt{{{{\rm{\gamma }}}_{{\rm{F}}}}^{{\rm{p}}}}(\frac{\sqrt{{{{\rm{\gamma }}}_{{\rm{L}}}}^{{\rm{p}}}}}{{{\rm{\gamma }}}_{{\rm{L}}}})$$

Equation (6) can be expressed as the following equation, where the values of ${{\gamma }_{F}}^{p}\,$and ${{\gamma }_{S}}^{d}$ are given by the tangent and y-intercept of Eq. (7) by the least square method:

7 $$\frac{(1+\,\cos \,\theta ){\gamma }_{L}}{2\sqrt{{{\gamma }_{L}}^{d}}}=\sqrt{{{\gamma }_{F}}^{p}}\cdot \sqrt{\frac{{{\gamma }_{L}}^{p}}{{{\gamma }_{L}}^{d}}}+\sqrt{{{\gamma }_{F}}^{d}}$$

The surface energy (*γ*_*F*_) of the Ag network/PTFE films can explain the sum that was calculated using Eq. (8):

8 $${\gamma }_{F}={\gamma }_{F}^{d}+{\gamma }_{F}^{p}$$

Table 1 shows the calculated value of the surface energy by polar and dispersive component and total surface energy, which is the summation of the polar component energy and dispersive component energy. Figure 3b shows that the PTFE coated Ag network electrode reveals much lower surface energy, compared to the bare Ag network film. At a thickness of 50 nm PTFE, the Ag network/PTFE shows a surface energy of 17.66 mJ/m^2^, indicating a super hydrophobic surface. The hydrophobic surface of the Ag network/PTFE film is favorable to the self-cleanable surface of the transparent heat-generating films for smart windows. Therefore, by simple sputtering of the PTFE layer on the Ag network, we can fabricate hydrophobic Ag network based thin film heaters, as shown in Fig. 3c. Water droplets on the surface of the Ag network/PTFE flew down fast, due to the hydrophobic surface of PTFE. This hydrophobic surface is desirable for self-cleanable smart windows in building and automobile windows.

**Table 1:** Surface energy of the bare Ag network and Ag network/PTFE films with increasing PTFE thickness.

Thickness of PTFE [nm]	DI-Water [mJ/m^2^] (Polar)	Diiodomethane [mJ/m^2^] (Dispersive)	Surface energy [mJ/m^2^]
0 (Bare)	12.28	32.17	44.45
5	0.69	22.57	23.26
10	0.45	25.21	25.66
15	0.22	20	20.23
20	0.19	19.04	19.24
50	0.15	17.51	17.66

**Figure 4:**
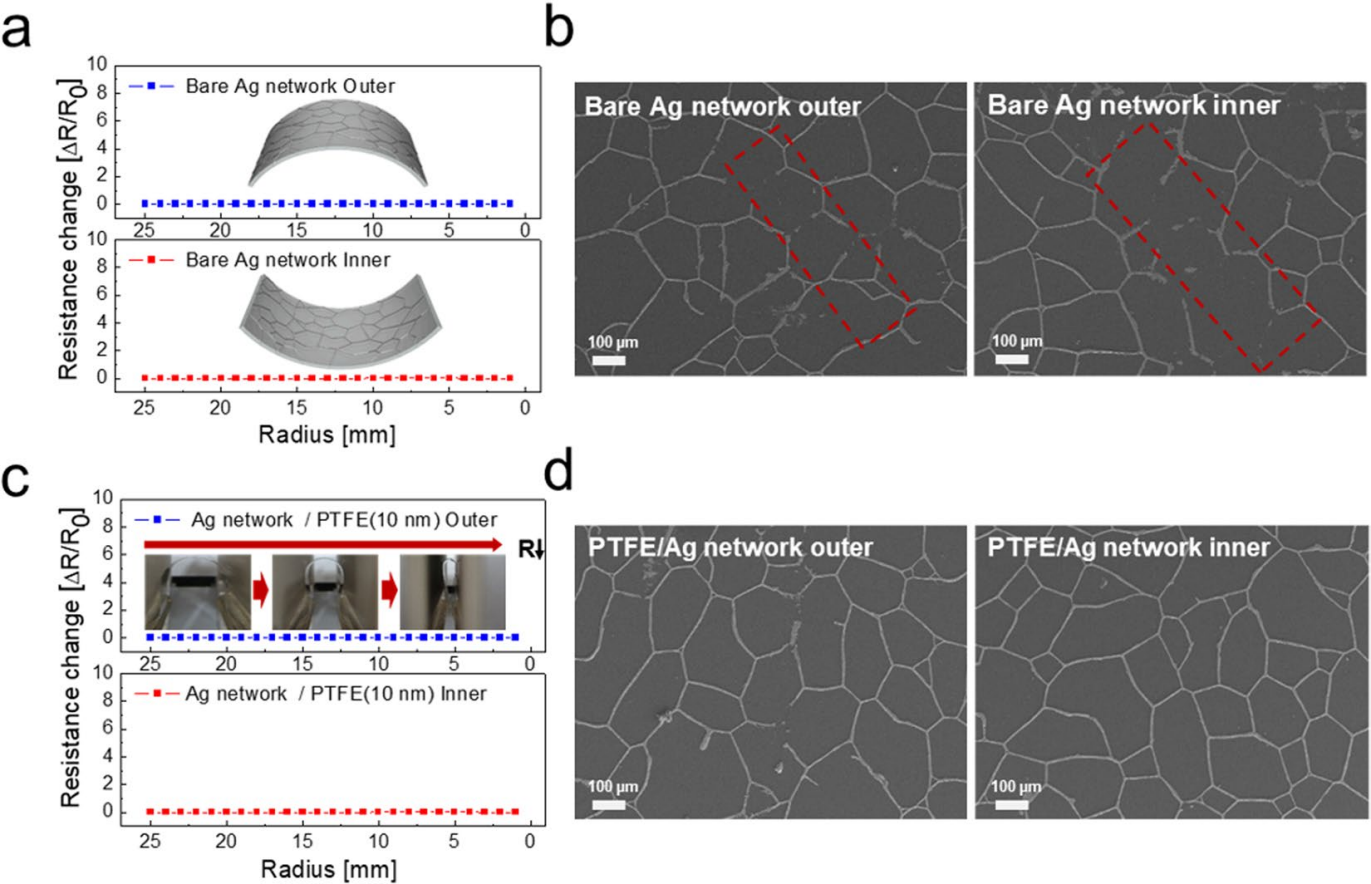
(**a**) Resistance changes of the bare Ag network films with decreasing bending radius. Inset shows the outer and inner bent sample. (**b**) Surface FESEM images of the bare Ag network after outer and inner bending below the bending radius of 1 mm. The dashed line indicates the delaminated Ag network during the outer/inner bending tests. (c) Resistance changes of the Ag network/PTFE (10 nm) film with decreasing outer and inner bending radius. The inset pictures show the outer bending steps in the specially designed bending system. (**d**) Surface FESEM images after outer/inner bending below the bending radius of 1 mm.

The mechanical properties of the Ag network/PTFE multilayer films were evaluated using a lab-designed bending test system. Considering curved glass or the specifically shaped surface of glass, the flexibility of TTFHs is very important. Figure 4a shows the outer and inner bending test results of the bare Ag network with decreasing bending radius. In the bending radius test, we measured the change of resistance (ΔR = (R-R_0_)/R_0_) by clamps, which tightly gripped the sample. R and R_0_ indicate the measured resistance and initial resistance of the electrode, respectively. In the case of the bare Ag network electrode in Fig. 4a, regardless of the outer and inner bending mode, the sample showed constant resistance change with decreasing bending radius from (25 to 1) mm. Due to the well-connected Ag network, the bare Ag network showed constant resistance change, even at the bending radius of 1 mm. However, we can observe the delamination of the Ag network at the severely curved region, as shown in the surface FESEM images in Fig. 4b. The dashed line indicates the delaminated Ag network at a severely curved region. The Ag network/PFTE (10 nm) films also show constant resistance change with decreasing outer and inner bending radius, as shown in Fig. 4c. However, even after the outer and inner bending radius of 1 mm, there is no delamination or cracks of the Ag network covered by PTFE layer (Fig. 4d). The sputtering of the PTFE layer could prevent the delamination of the Ag network, due to the outstanding flexibility of the polymer PTFE layer.

**Figure 5:**
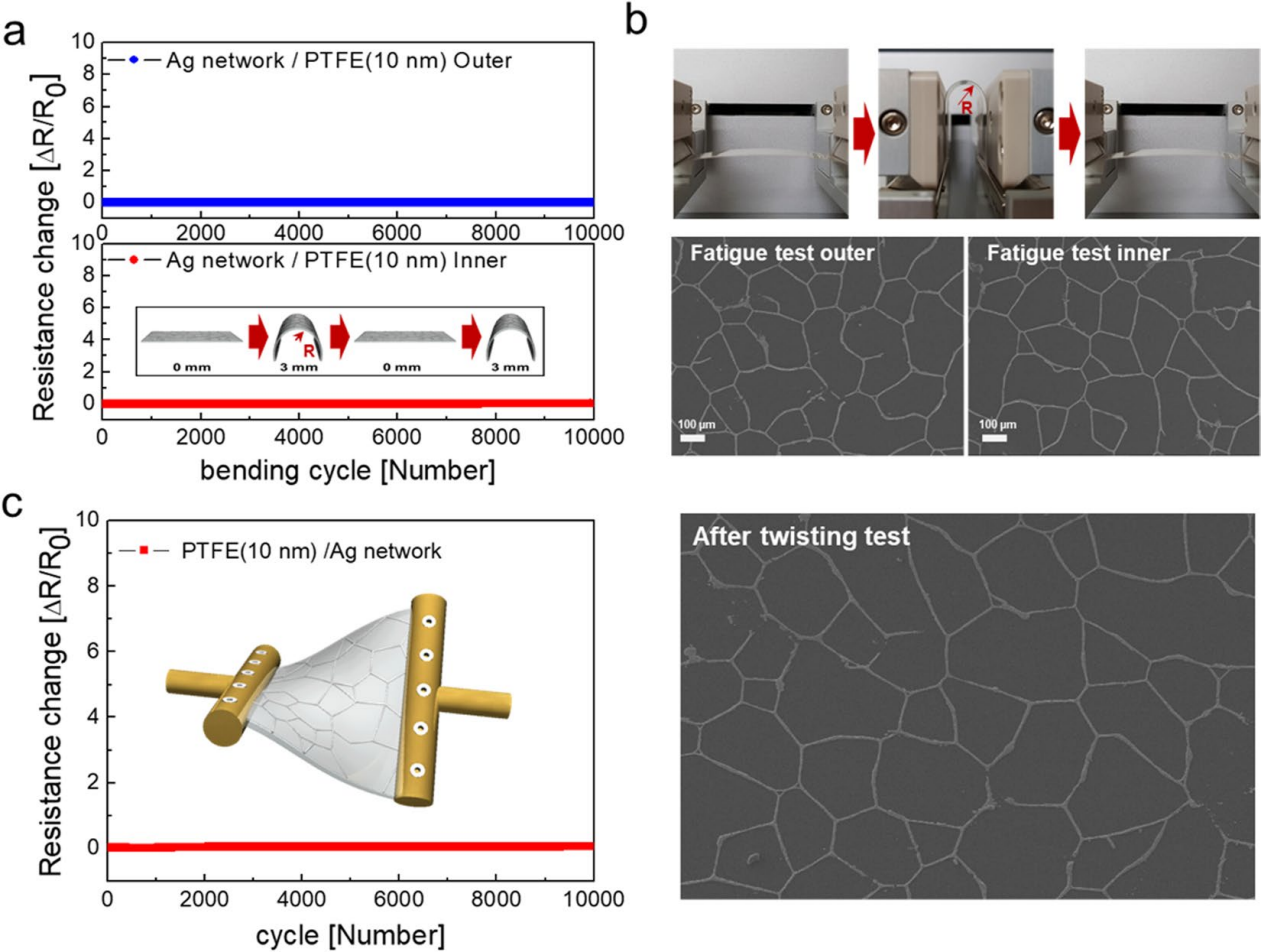
(**a**) Resistance change of the dynamic fatigue bending test of the Ag network/PTFE (10 nm) films at a fixed outer and inner bending radius of 3 mm for 10,000 cycles. Inset shows the schematics of the dynamic fatigue steps. (**b**) Pictures of the dynamic fatigue bending test; by pressing the plates on both sides, the bending radius of a sample can be exactly controlled, and surface FESEM images are shown of the Ag network/PTFE films after 10,000 times repeated outer and inner bending at a constant bending radius of 3 mm. (**c**) The Resistance change during the repeated twisting test for 10,000 cycles at a constant twisting angle of 20°, and surface FESEM image of the network after the test.

To show the mechanical reliability and durability, the dynamic outer and inner bending test for 10,000 cycles was carried out. The sample was bent to bending radius of 3 mm, and extended to 0 mm repeatedly, as shown in the inset. Figure 5a shows the resistance change of the Ag network/PTFE films as a function of the repeated bending cycles for 10,000 times at a constant outer and inner bending radius of 3 mm. For 10,000 cycles, the Ag network/PTFE films showed a constant resistance change, due to the mechanical flexibility of the Ag network covered by PTFE layer. After 10,000 cycles, the Ag network/PTFE films showed identical surface morphology to the as-fabricated sample, as shown in Fig. 5b. Outstanding mechanical flexibility of the Ag network/PTFE films is desirable for the transparent thin film heater to attach to the curved surface of smart windows. Figure 5c also demonstrates the twisting test results for Ag network/PTFE films by using our lab-designed twisting test system. There was no change of resistance during repeating twisting for 10,000 cycles. Based on the bending and twisting test of the Ag network/PTFE films, we confirmed the outstanding mechanical flexibility of the Ag network/PTFE films for curved or specially shaped smart windows.

**Figure 6:**
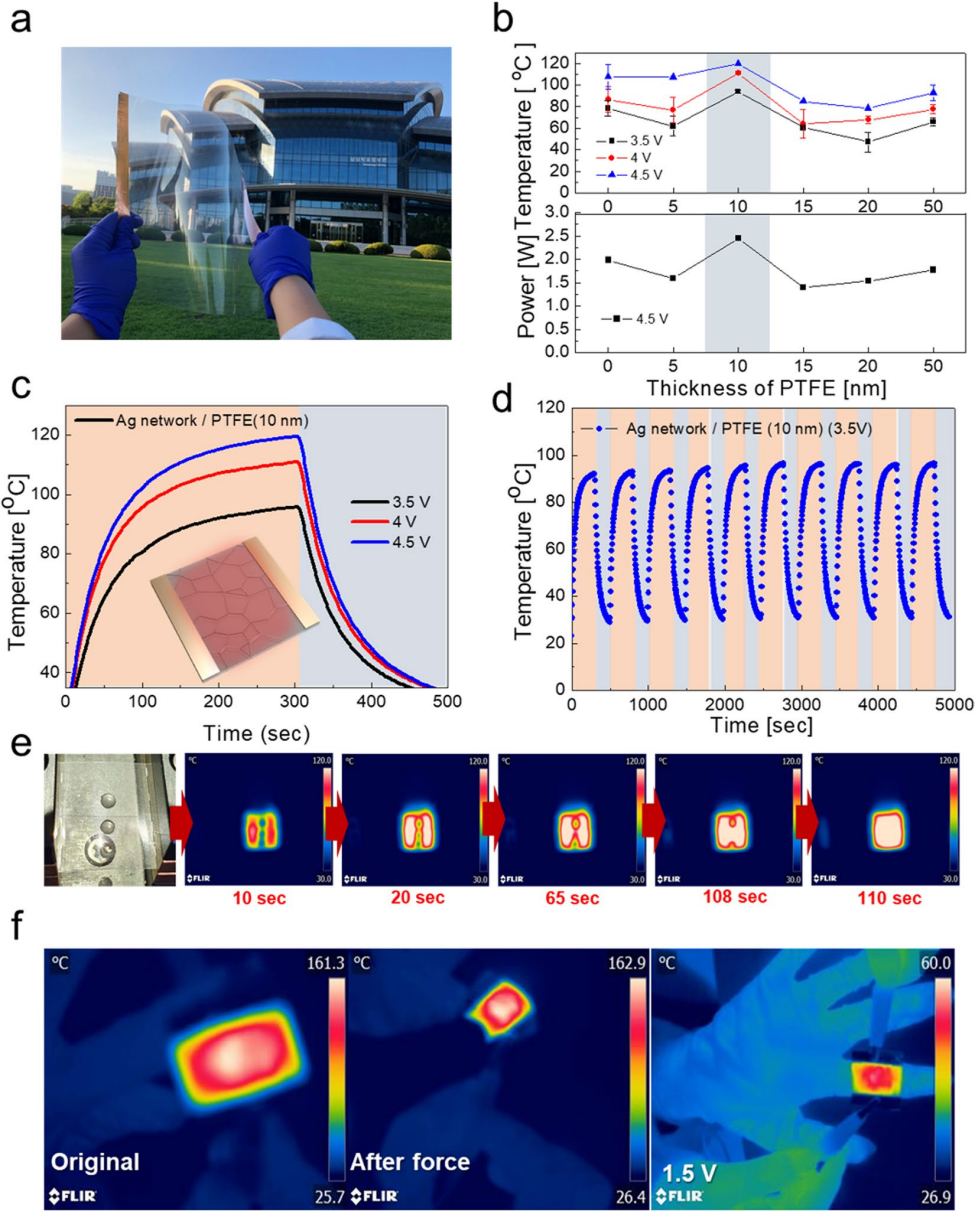
(**a**) Large size of heat generator using Ag network/PTFE films. (**b**) Saturation temperature and power of heat generator using Ag network/PTFE films with increasing PTFE thickness of (0 to 50) nm. (**c**) Temperature profiles with increasing voltage of (3.5 to 4.5) V, and (**d**) repeated heating and cooling cycles at 3.5 V during 5,000 s of the Ag network/PTFE films-based heat generator. (**e**) IR images of the evaporation of water droplets on the hydrophobic heat generator. (**f**) IR images of the flexible heat generator before and after a given force and attached to a human finger as a wearable heat generator.

To demonstrate the feasibility of the Ag network/PTFE film as a multi-functional film for smart windows, we fabricated flexible TTFHs Ag network/PTFE electrodes. The flexible TTFHs had edge contact electrodes (Cu tape or Ag paste) to apply power for heat generation. Figure 6a shows a picture of the large-area flexible TTFHs fabricated on Ag network/PTFE films with Cu edge electrodes. Figure 6b (upper panel) shows the measured temperature of the flexible TTFHs with Ag network/PTFE film with different PTFE thickness by thermocouples directly mounted on the TTFHs. With increasing input voltage from (3.5 to 4.5) V, the saturation temperature of flexible TTFHs increased. Regardless of the PTFE thickness, the saturation temperature and power of all the TTFHs increased with increasing input voltage. Due to the similar sheet resistance, both bare Ag network and Ag network/PTFE based TTFHs showed similar saturation temperature at a constant input voltage. As expected from Fig. 2a, all TTFHs showed similar saturation temperature at the same input voltage, since the main conduction path and heating source in the Ag network/PTFE films is the well-connected Ag network. As we discussed in regard to the thin metal based thin film heater, air convection is the main path of heat dissipation in the Ag network/PTFE films-based flexible TTFHs^38^.

9 $${Q}_{conv}=\frac{{V}^{2}}{R}\varDelta t={h}_{conv}{A}_{conv}({T}_{s}-{T}_{i})$$

10 $${T}_{s}=\frac{{V}^{2}\varDelta t}{R{h}_{conv}{A}_{conv}}+{T}_{i}$$

Here, hconv is the convective heat transfer coefficient, Aconv is the surface area, and Ts and Ti are the saturation and initial temperature, respectively. These equations make it clear that the saturation temperature of flexible TTFHs increases with increasing input DC voltage (V), and with decreasing resistance (R). Although all the Ag network/PTFE electrodes showed similar sheet resistance, the TTFH with Ag network/PTFE (10 nm) showed the highest saturation temperature of greater than 90 °C at all input voltage, and highest power at the constant input voltage of 4.5 V. Due to the passivation effect of thermal energy, the TTFH with 10 nm thickness of PTFE coated Ag network films showed higher saturation temperature than the bare Ag-network. However, further increase in PTFE thickness led to decrease in saturation temperature, due to the insulating properties of the thick PTFE layer covering the Ag network. Figure 6c shows the temperature profiles of the flexible TTFT using the optimum PTFE (10 nm)/Ag network films. The temperature of the flexible TTFH increased with increasing DC voltage from (3.5 to 4.5) V, as expected from Eq. (9). At 4.5 V applied voltage, during the turn-on term (300 s), the flexible TTFH reached the highest temperature of 120 °C. In addition, Fig. 6d shows the repeated temperature profile of TTFH during repetitive increase and decrease of the temperature for 10 times by repeatedly applying an operating voltage of from (0 to 3.5) V. The saturation temperature is maintained above 90 °C during 10 cycles. The result shows the flexible TTFH had good thermal stability, due to the effective passivation of the sputtered PTFE layer. Figure 6e shows that we dropped water onto the Ag network/PTFE based TTFH. The water droplets had a high contact angle, due to the hydrophobic surface of the Ag network/PTFE films. The hydrophobic TTFHs could provide the self-cleaning function on the smart window, as well as heat generation. These multi-functional films are desirable for next generation building and automobiles. After dropping of the water, we applied input voltage of 4.5 V to the TTFH to evaporate water droplets. The water droplets gradually became smaller over time. At 110 sec, all water droplets had evaporated from the TTFH. Due to the water droplet evaporating in only 110 sec, the surface of the Ag network/PTFE films can quickly come back to a clean state, without water stain. Furthermore, the Ag network/PTFE-based TTFH can be used as a wearable heating patch for human, because the Ag network/PTFE has outstanding flexibility. Figure 6f shows IR images of the flexible TTFH before and after curving of the sample. Even at the severely curved state, the TTFH showed constant temperature, due to the outstanding flexibility of the Ag network/PTFE films. In addition, the left side IR image demonstrates that the TTFH wrapped around the finger operated well after applying a low voltage of 1.5 V, and could act as a wearable heat patch for human.

**Figure 7:**
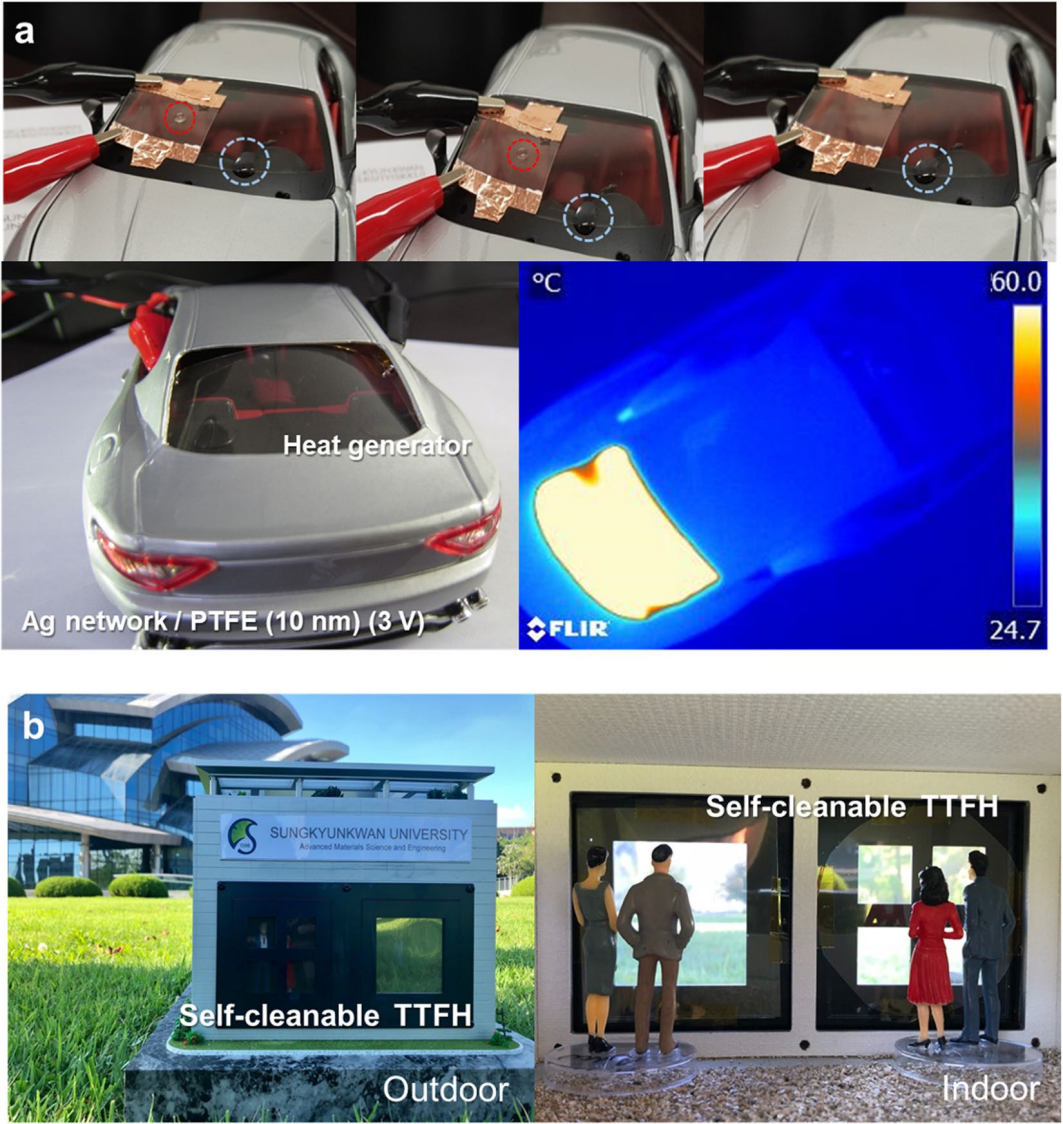
(**a**) Upper images show comparison between evaporation of water droplets onto the hydrophobic heat generator based on Ag network/PTFE films, and the original window. IR images show the heat generator operating at 3 V used for rear windows. (**b**) Promising applications of the self-cleanable TTFH as smart windows for buildings.

Figure 7 demonstrates the promising application of Ag network/PTFE films as smart windows. The Ag network/PTFE electrode-based flexible TTFHs can be use in smart windows, especially in automobiles and buildings, as shown in Fig. 7. Since the optimum Ag network/PTFE films had a high transmittance of 80.0% at a wavelength of 550 nm and a super hydrophobic surface, it is acceptable as a front window for automobiles. The upper panels in Fig. 7a compare the wettability of the water droplet on the Ag network/PTFE and bare Ag network. The optimum Ag network/PTFE films based TTFHs showed good performance at low voltage and hydrophobic surface. The water droplet on the TTFH with Ag network/PTFE electrode could be rapidly thrown away when the automobile moves, due to the hydrophobic surface of the Ag network/PTFE films. In addition, frost on the window is easily removed by the effective heating of Ag network/PTFE electrodes. Due to the curved surface of the rear window in automobiles shown in Fig. 7a, the TTHS for automobiles should have good flexibility and high transparency. For this reason, the frost cannot form on the Ag network/PTFE based-smart windows, so the visibility can be significantly improved during driving, even at a temperature below zero. Moreover, the Ag network/PTFE based-self-cleanable TTFHs could be employed as outdoor windows in smart buildings, to remove the frost on the surface of smart windows, as shown in Fig. 7b.

## Discussion

Highly transparency and flexibility Ag network films were fabricated by a bar-coating system on pre-treated PET substrate. To use as smart windows to be built into automobiles and buildings, we sputtered PTFE onto Ag network films. In spite of the coating of PTFE layer, the optical transmittance and sheet resistance of the Ag network/PTFE films were not affected by the thickness of the PTFE layer. In addition, the Ag network/PTFE films had hydrophobic surface, because the PTFE layer had low surface energy, caused by the strong chemical bonding between carbon and fluorine. In addition, the Ag network/PTFE films showed good mechanical flexibility, and no resistance change during outer/inner bending and twisting tests. According to the FOM values, we optimized the PTFE thickness as 10 nm in the Ag network/PTFE films, due to the low sheet resistance and high optical transmittance, as well as the hydrophobic surface, which are requirements of the flexible TTFHs. The flexible TTFHs fabricated on the optimum PTFE (10 nm)/Ag network films showed a high saturation temperature of 120 °C, even at a low input voltage and power. In addition, due to the hydrophobic surface of the PTFE coating layer, the TTFH with Ag network/PTFE films can maintain a clean surface, even after water dropping. Due to the effective heat generation of the Ag network and the hydrophobic surface of the PTFE layer, the Ag network/PTFE hybrid films could be used as multi-functional electrodes for flexible TTFFs, which are attached to smart windows in buildings and automobiles.

## Methods

### Fabrication of the Ag network film on flexible PET substrates

The wide Ag network films (680 mm) were prepared on flexible polyethylene terephthalate (PET) substrates by a bar-coating system (KP-3000VH, Kipae E&T Co.). To coat the Ag network films, we prepared PET substrate with low surface energy by surface treatment (DOF Co.) and Ag nanoparticle solution. The Ag nanoparticle solution was made by mixing 54 wt.% of TCC A with 46 wt.% of TCC B solution, and sonicated 3 times for 30 s to homogenize. The Ag nanoparticle solution was covered onto pre-treated PET substrate, and the bar of the bar coating system was scanned at 30 mm/sec. After coating, the films were dried at 50 °C for 1 min and continually at 150 °C for 1 min 30 s to make self-assembled embossed-type Ag network films.

### Sputtering PTFE layer on Ag network films

The polytetrafluoroethylene (PTFE) layer was coated onto Ag network films using an MF magnetron sputtering system at room temperature. To sputter the PTFE layer, we prepared 4-inch PTFE (A7-J, Dupont Mitsui, 97 wt.%) and CNT (HANOS CM-280, Hanwha Chemical, 3 wt.%) mixed conductive target (Jaewooenpla Co.), and the target was loaded on dual cathode gun, and sputtered the target at a constant MF power of 150 W (power density 3.29 W/cm^2^), an Ar flow of 50 sccm, and a working pressure of 9 mTorr. We stacked the increasing thickness of PTFE from (5 to 50) nm. The thickness of each layer was confirmed by surface profiler (Alpha-step 250, Tencor).

### Analysis of the Ag network/PTFE films

The electrical and optical properties of the Ag network/PTFE films were measured using a four-point probe (FPP-HS8, DASOL ENG), Hall-measurement (HL5500PC, Accent Optical Technology), and UV/Visible spectrometry (V-670, Jasco). The mechanical properties of the Ag network/PTFE films were investigated by our lab-designed inner/outer bending test system and twisting test system. To confirm the mechanical stability, we fixed the bending radius of 3 mm of dynamic fatigue bending test for 10,000 bending cycles and a twisting angle of 20°. The surface of the Ag network/PTFE films were analyzed by field emission scanning electron microscopy (JSM-7600F, JEOL) and surface morphology of the bare Ag network and Ag network/PTFE films were investigated by atomic force microscopy (AFM, XE-100, Park system). To calculate the surface energy of the Ag network/PTFE films, we investigated by contact angle measurements (Phoenix-MT(A), SEO CO.), using the polar and dispersion components of deionized water and diiodomethane with a constant volume of 3 ml.

### Fabrication and evaluation of smart windows (heat generator)

To confirm the possibility of using the Ag network/PTFE films for flexible smart windows, we fabricated a heat generator with a size of (25 mm × 25 mm). To fabricate the heat generator, Ag paste was brushed onto the two terminal sides of the Ag network/PTFE films, and Cu tape was attached on the Ag paste. DC voltage was supplied by a power supply (OPS 3010, ODA technologies) to the heat generator using Ag network/PTFE films through a Cu/Ag paste contact electrode. The temperature and thermal stability of the heat generator were measured using a thermocouple mounted on the surfaces of the heat generator and an IR thermal imager (A35sc, FLIR).

### Manuscript comments

One of the co-author (Ji-Eun Lee) drew drawings in Fig. 1a, inset of Fig. 3b insets of Fig. 4a, insets of Fig. 5a, insets of Fig. 5c, and insets of Fig. 6c using a **RHINO** drawing program. The url link of the **RHINO** drawing program is http://www.rhino3d.com. In addition, we checked the terms of use of the software **RHINO**.
